# Dissolution of β-C_2_S Cement Clinker: Part 1 Molecular Dynamics (MD) Approach for Different Crystal Facets

**DOI:** 10.3390/ma15186388

**Published:** 2022-09-14

**Authors:** Khondakar Mohammad Salah Uddin, Mohammadreza Izadifar, Neven Ukrainczyk, Eduardus Koenders, Bernhard Middendorf

**Affiliations:** 1 Department of Structural Materials and Construction Chemistry, University of Kassel, Mönchebergstraße 7, 34125 Kassel, Germany; 2 Institute of Construction and Building Materials, Technical University of Darmstadt, Franziska-Braun-Str 3, 64287 Darmstadt, Germany

**Keywords:** cement dissolution, belite clinker C_2_S, free energy surfaces, crystal facets and defects, molecular dynamics simulation, ReaxFF, metadynamics, atomistic activation energy

## Abstract

A major concern in the modern cement industry is considering how to minimize the CO_2_ footprint. Thus, cements based on belite, an impure clinker mineral (CaO)_2_SiO_2_ (C_2_S in cement chemistry notation), which forms at lower temperatures, is a promising solution to develop eco-efficient and sustainable cement-based materials, used in enormous quantities. The slow reactivity of belite plays a critical role, but the dissolution mechanisms and kinetic rates at the atomistic scale are not known completely yet. This work aims to understand the dissolution behavior of different facets of β-C_2_S providing missing input data and an upscaling modeling approach to connect the atomistic scale to the sub-micro scale. First, a combined ReaxFF and metadynamics-based molecular dynamic approach are applied to compute the atomistic forward reaction rates (R_D_) of calcium (Ca) and silicate species of (100) facet of β-C_2_S considering the influence of crystal facets and crystal defects. To minimize the huge number of atomistic events possibilities, a generalized approach is proposed, based on the systematic removal of nearest neighbors’ crystal sites. This enables us to tabulate data on the forward reaction rates of most important atomistic scenarios, which are needed as input parameters to implement the Kinetic Monte Carlo (KMC) computational upscaling approach. The reason for the higher reactivity of the (100) facet compared to the (010) is explained.

## 1. Introduction

A huge production of Portland cement (PC) needed to satisfy the demand of construction industry is responsible for approximately 5% of global anthropogenic CO_2_ emission [1,2,3]. Complying with future climate regulations asks for a massive reduction in the cement clinker content, leading to the so-called eco-concretes. Therefore, the production of cement clinkers with a lower CO_2_ footprint and improved hydration performance is the key challenge to develop climate-friendly concretes. For instance, the production of belite (C_2_S) is less energy intensive (25% lower) [4] and emits 10–15% less CO_2_ compared to alite (C_3_S) [5,6]. However, its lower reactivity remains a major issue that needs to be addressed. Increasing the reactivity of C_2_S is an alternative way to produce more environmentally friendly and relatively cost-effective C_2_S-based cement [6].

Cement hydration is a complex reaction process, involving the concomitant reaction of many mineral phases. Therefore, in order to better understand the early stage of hydration, much research has been conducted considering the alite reaction [7,8]. However, the dissolution behavior of several anhydrous crystal facets of belite is still unknown. Dissolution-precipitation processes are acting simultaneously during the different stages of cement hydration. More specifically, the measured net hydration rate (e.g., by a calorimeter) is a result of the chemical coupling between the following two simultaneous reactions: (1) the clinker dissolution and (2) the hydrates (e.g., C-S-H and portlandite) precipitation. Thus, identifying which of the two reaction mechanisms is the rate-limiting kinetic step is of major importance, and can only be attained by studying those two reaction mechanisms separately. Recently, Naber et al., [9] demonstrated that measured reaction rates of alite hydration during the acceleration stage are well predicted by theoretical rates calculated from C-S-H precipitation, at a wide range of water/cement mass ratios (w/c = 0.5…10). This implies that a correct kinetic description of C-S-H precipitation is already available. However, a theoretical modeling approach to predict dissolution rates is still missing. The knowledge about the dissolution (decoupled from precipitation) of different facets of C_2_S is essential to understanding and predicting the hydration mechanism. Belite is the second most important clinker phase (15–30 wt.%) in OPCs after alite (C_3_S). Five different polymorphs (α, $\alpha_{H}^{\prime }$, $\alpha_{L}^{\prime }$, β and γ) are identified by thermal and X-ray measurements at normal pressure; nevertheless, β-C_2_S is substantially more reactive compared to other variants [1,10]. The molecular process of cement clinkers dissolution, especially at an atomistic scale, is not fully understood yet. Molecular dynamics (MD) has been a very efficient method to explore the interfacial reaction mechanism of the crystals at an atomistic scale [11]. 

This paper focuses on the atomistic models for the dissolution of β-C_2_S crystals and develops an elementary physical/chemical bridging model for the initial dissolution of C_2_S that proposes an approach to connect the nanoscale to the upscaled microscale level. MD simulations are indomitable tools for revealing how the atomistic dissolution events of individual crystal sites affect the upscaled dissolution kinetics. However, the major challenge is in upscaling, i.e., how to statistically consider the huge number of individual atomistic events for individual crystal sites, which, moreover, depend on many neighboring scenarios (e.g., crystal defects, kink, step retreat effects). To be able to close such a huge gap between the atomistic and the meso-scale modeling approaches for dissolution of clinker crystals, first, the focus has to be put on the fundamental forward dissolution rate constant, i.e., by considering the far-from-equilibrium conditions, where effects of precipitation and concentration of dissolved species can be neglected. Many computational methods have been developed to calculate the reaction mechanism of cementitious materials at an atomistic scale [12,13]. Although density functional theory (DFT) is considered to be the best for accurate atomistic description, due to its high computational cost and limitation to the small number of atoms, it is inapplicable for large crystals [14,15,16]. In contrast, the classical force field lacks the capability to calculate the reactions. Therefore, to bridge the gap between the force field theory and quantum mechanical calculations, ReaxFF has been developed to simulate molecular dynamics in large-scale chemical systems (size in the order of thousands of atoms) successfully for investigating the reaction mechanism at the material interface with reasonable computation time. It is almost 2000 times computationally cheaper than the DFT. It has already been implemented successfully in hydrocarbons [17], polymer chemistry, metal oxides (Si/SiO_2_) [18,19,20], and many other systems [21]. ReaxFF uses the parameter set derived by DFT, consequently, the accuracy of this method is comparable to DFT calculation with the deviation of 2 Kcal/mol during the energy calculation. 

ReaxFF has been an efficient method, however, calculating the transition state (TS) of a reaction using ReaxFF becomes computationally expensive due to its femtoseconds (10^−15^ s) time steps calculation approach. Therefore, MetaD was combined with ReaxFF to minimize the computational cost. The combined approach has already been implemented to calculate the dissolution mechanism and the reactivity of different facets of Portlandite at an atomistic scale [22]. 

This paper (Part 1) reports a multistep modeling approach used to get an insight into the initial dissolution and interfacial reactivity of C_2_S facets at room temperature (298 K). The first part provides the pre-simulations of (001), (100), and (010) facets of C_2_S, for 600 picoseconds to allow for pre-hydration. The reactivity difference between the facets was investigated from the dissolution profile (free energy surface) of calcium atoms from each pre-hydrated facet using ReaxFF coupled with MetaD [23]. The second part focuses on the detailed study of the different scenarios of the (100) facet of C_2_S which leads to many possibilities and the variation in dissolution rates of calcium and silicates due to the removal of the neighbors [22]. The MD calculations enabled to obtain activation energies for the identified atomistic events, which can be used to calculate the atomistic rate constants employing a transition state theory. The influence of neighbors on the reaction rate of individual scenarios at an atomistic scale is identified as the key step for upscaling to the mesoscale simulation. Therefore, the calculated atomistic activation energies (proportional to forwarding reaction rates according to TST) of possible events depending on the neighboring scenarios are provided as the required input parameters for the Kinetic Monte Carlo (KMC) upscaling approach (in Part 2 [24], contributed within this issue) for the computation of the mesoscopic forward dissolution rates of (100) facet of C_2_S and microstructural evolution occurred during the dissolution process [25].

## 2. Methods and Modeling Approach

The employed ReaxFF parameter set (Si-O-H and Ca-O-H) has already shown a great potential to explain the mechanical properties of C-S-H and the dissolution mechanism during the early hydration of cement clinkers (C_3_S) [19,23,26]. MetaD is a powerful sampling algorithm that accelerates the TS observation by introducing a biased potential on a specified collective variable (CVs) [27]. It can act directly on the microscopic coordinate system during an MD simulation and reconstruct the free energy surfaces (FES) [27,28]. The entire simulations were carried out by using ReaxFF in the LAAMPS (Large-scale Atomic/Molecular Massively Parallel Simulator, version 2018) platform [29], and, the PLUMED package was added as an extension of LAMMPS to perform the MetaD simulations [30,31,32].

### Model Construction

β-C_2_S has a monoclinic crystal structure with the space group *P*2_1_/*n*_1_ [2]. A single crystal lattice contains 8 Ca, 4 Si, and 16 O atoms, where each Ca links to 6 oxygen to form (CaO_6_) octahedron, and Si forms a tetrahedron by covalent bonds with 4 O. In this research a β-C_2_S crystal model was used with the lattice parameter of a = 5.51 Å, b = 6.76 Å, c = 9.33 Å, α = γ = 90° and β = 94.17° [10,33]. The fresh cleaved orthogonal simulation cell of β-C_2_S containing (100) facet (21.90 × 27.04 × 18.56) × 10^−30^ m^3^ composed of 896 atoms was constructed by virtual nano lab (VNL) [34] and Avogadro [34,35] (Appendix A). After optimizing the geometry using HFTN (Hessian-free truncated Newton algorithm) considering the cutoff tolerances for energy and force 4.18 × 10^−4^ and 4.18 × 10^−8^ kJ mol^−1^, respectively [36], an additional 1.18 × 10^−26^ m^3^ periodic cell filled with water was added on the (100) facet of β-C_2_S using packmole [37]. In this upper compartment of the simulation cell, the total number of randomly distributed water molecules matched a density of 1000 kg m^−3^. Since calculating the macroscopic properties of the real system is required to consider a representative number of particles. Thus, MD simulations using ReaxFF should checked to use a representative number of particles. Moreover, ReaxFF calculates the movement of all atoms, therefore, a larger simulation cell increases the computational cost. To solve this time-space scaling issue, a periodic boundary condition was applied to mimic the infinite particle system and avoid atom loss during simulation. On the other hand, the simulation cell should not be too small to avoid unrealistic self-interaction, when a particle sees its image. Therefore, the dimension of the simulation cell should be higher than twice the cut-off radius (4.5 Å). In addition, the reactive surface must be large enough to get a representative picture of the atomic rearrangement. First, the simulation cells were equilibrated to 298 K and 1 atm for 150 picoseconds with 0.5 femtoseconds time steps using a Nose−Hoover thermostat (NVT ensemble). Afterward, it was hydrated for 600 picoseconds using Nose−Hoover barostat (NPT ensemble) with all three diagonal components of the pressure tensor to be coupled together (iso). The last geometry of the pre-hydrated (100) facet of C_2_S (after 600 picoseconds) was selected to calculate the dissolution mechanism using the combined approach of MetaD and ReaxFF. 

A calcium Ca-744 from (100) facet placed in between two silicates is selected to calculate the dissolution mechanism from facet to solution using a well-tempered MetaD scheme. The distance between the center of mass (COM) of the crystal and the specified calcium atom is selected as a CVs and biased potential was added as a Gaussian with frequency 40. Furthermore, Gaussian hills, having a height of 6.28 kJ /mol and a full width at half-maximum of 0.2 × 10^−10^ m are added every 0.02 picoseconds. The MetaD-ReaxFF simulations were run for 500 picoseconds (until converged) with NPT ensemble at temperature 298 K, and the total free energy of dissolution was calculated. 

A similar approach was used for other crystal facets: (001), (010). The reactivity of the individual atomistic events was calculated from the free energy of activation and the total free energy change. 

**Figure 1:**
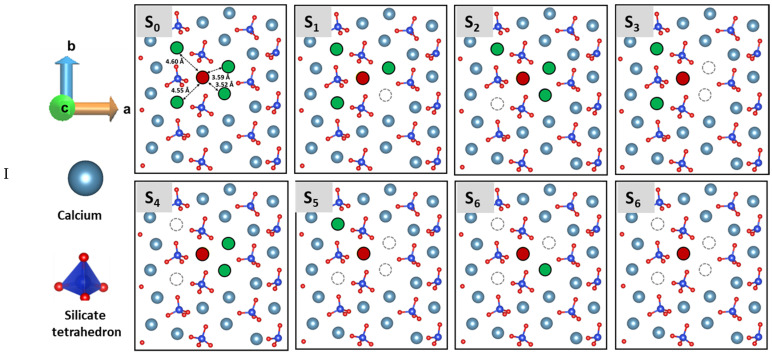

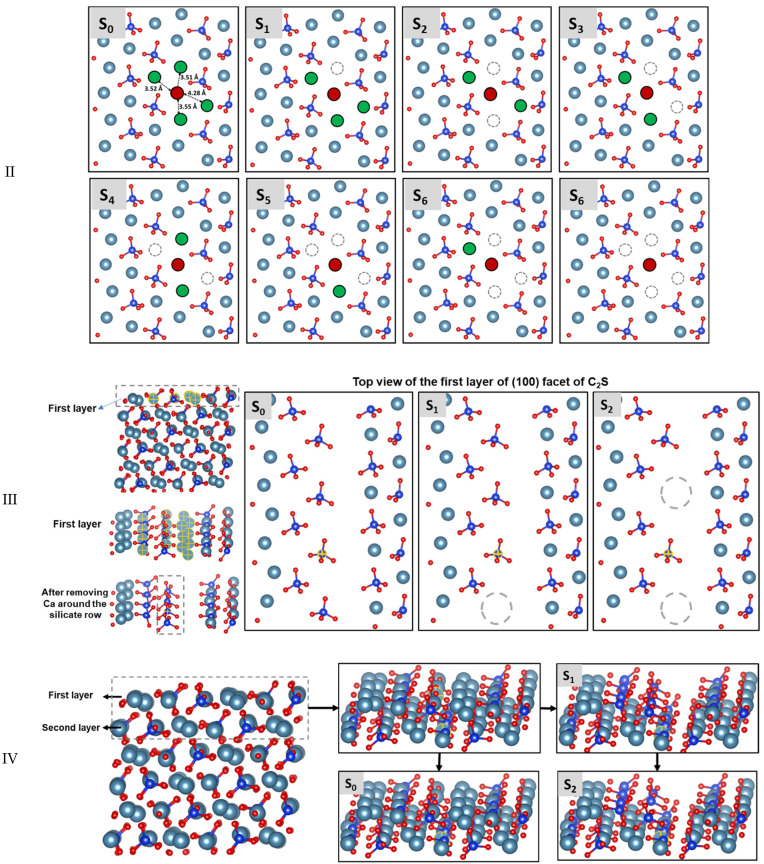
Dissolution scenarios of (**I**) the central Ca (marked red) located in between two silicates (Ca-SiO_4_ row); (**II**) the central Ca (red) located in the Ca layer from (100) facet of C_2_S in absence of 1, 2, 3 and 4 Ca neighbors (green) removed at different combinations (scenarios) for both cases (S_0_–S_6_). (**III**) Dissolution scenarios of central silicate (marked with yellow cross), after stepwise removal of the surrounding Ca and in the presence (a) and absence (b,c) of the two nearest silicate neighbors located in a row. (**IV**) Dissolution behavior of a central Ca (marked yellow cross) located in between two silicates in the second layer (S_1_; stepwise removal of Ca nearest neighbors (within 5 Å distance) and (S_2_) one silicate from the first layer.

In order to understand the overall dissolution of C_2_S (100) facet at higher time and space scales, the influence of both Ca and silicate neighbors on the dissolution process are computed, and made available as an input table for upscaling by KMC approach. Additionally, the molecular arrangement of (100) facet of C_2_S has indicated two different types of Ca. First, (Case. I) located between silicates (Ca-SiO_4_ row) and second, (Case. II) in between two Ca (Ca row). Therefore, two cases with different scenarios were considered by selecting each type of Ca as a center and 4 Ca-neighbors located around the 5 Å radius of the center. A similar modeling approach was applied to compute the dissolution profile of central Ca (red) in absence of 1,2,3,4 Ca neighbor (Green) being pre-deleted (and equilibrated) in different combinations (Figure 1I,II). In this manner, the activation barrier for a total of 8 scenarios was calculated for each case to investigate the influence of neighbor (scenario) configurations that are expected to occur during the (longer-term) dissolution. 

Furthermore, the dissolution of Ca^2+^ ions is more probable compared to silicate due to their strong interaction with neighboring Ca. Therefore, firstly, all Ca atoms are removed to free the silicate row. Hence, a similar modeling approach is applied to calculate the dissolution profile of the central silicate (marked yellow cross) before and after removing 1 and 2 neighboring silicates located on both sides in the same row (Figure 1III).

Moreover, investigating the possibility of etch pit formation, the dissolution behavior (FES) of selected Ca (marked yellow cross) from the second layer was calculated in the presence and absence of Ca, and the silicate of the upper layer (Figure 1IV).

Finally, this intensive study brings a set of most important events for the (100) facet of C_2_S. According to the transition state theory (TST) [38,38], the calculated activation barrier based on the MD simulation is proportional to the individual rate constant (using a TST equation [38]). This enables to deliver of a set of individual reaction rates, as a basic input for upscaling the KMC approach to calculate the overall meso-scopic dissolution rate of (100) facet of C_2_S as well as investigate morphological changes during dissolution (published separately as part two contribution to this issue) [39].

## 3. Results and Discussion

### 3.1. Pre-Hydration of β-C_2_S

β-C_2_S is responsible for the strength development of concrete at a later age due to its lower reactivity, however, compared to C_3_S it precipitates different ratios of similar hydration products, C-S-H (calcium silicate hydrate) and portlandite [23]. The knowledge of the dissolution behavior of different facets of β-C_2_S could lead to an in-depth understanding of their reactivity toward hydration. Therefore, the interaction between the facets of β-C_2_S and water bulk was investigated by following the reaction dynamics, first for 600 picoseconds.

**Figure 2:**
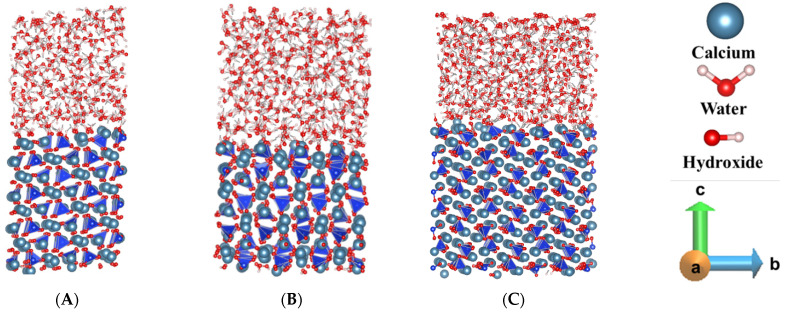
Representative snapshot for comparing the reactivity between the β-C_2_S facets: (**A**) (100), (**B**) (001), (**C**) (010) after pre-hydration for 600 picoseconds at 298 K.

Generally, β-C_2_S has shown less reactivity due to its compact crystal structures and the absence of free oxygen as compared to the C_3_S, since the free oxygen of C_3_S is an influential factor that leads to a strong interaction with bulk water [11,23,40]. According to our observation, comparing all the pre-hydrated crystal facets of β-C_2_S, (100) and (001) has shown higher reactivity towards hydration (Figure 2A,B), however, the interactions were not strong enough to show air void formation as observed in C_3_S facets [11]. In contrast, the (010) facet of β-C_2_S has shown significantly lower reactivity than the other two facets (Figure 2C). The reactivity difference between the facets was further elucidated through the dissolution mechanism of Ca located in between two silicates of the respective facets. The free energy profile of the Ca dissolution has explained the reactivity by comparing the activation barrier and free energy change during the dissolution process.

### 3.2. Dissolution of Calcium from (100), (001), and (010) Facets of β-C_2_S

**Figure 3:**
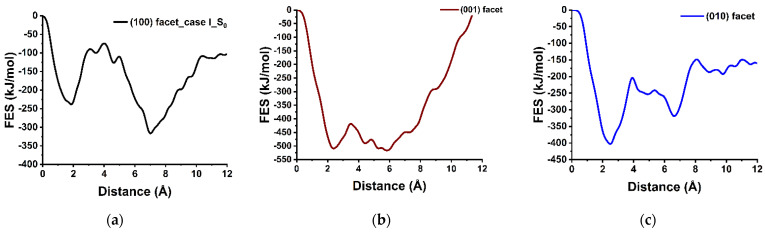
The representative dissolution profile is quantified by free energy surface (FES) of Ca from: (**a**) (100), (**b**) (001), (**c**) (010) crystal facet of β-C_2_S at 298 K.

Free energy calculation (MetaD) has been used in MD simulations for finding the reaction pathways including transition states (TS). In the MetaD calculation, a single CV (the distance between the Ca-548 and the center of mass of the crystals) was selected to obtain FES. The *x*-axis and *y*-axis denote the reaction coordinate in terms of distance in Å (10^−10^ m) and FES in terms of energy in kJ/mol, respectively. The activation barrier for Ca-548 dissolution was calculated considering the first minima (initial position) and highest peak (TS) of the FES scanning over the range of 0 to 12 Å. Figure 3a indicated the movement of Ca-548 from the (100) facet to the solution by overcoming the activation barrier of 164.30 kJ/mol at 4.20 × 10^−10^ m, after a small fluctuation, it was completely dissolved, moving into the solution by 7.20 × 10^−10^ m. The process was indicated thermodynamically favorable and exergonic, from the value of total free energy change (Δ*G*) of −78.00 kJ/mol at 298 K. Similarly, the (001) facet is also reactive as well, however, overcoming the lower activation barrier of 90.80 kJ/mol compared to the (100) facet (Figure 3b vs. Figure 3a). The results support the trend that was predicted after pre-hydration. Moreover, the (001) facet of C_3_S was found less reactive and thermodynamically unfavorable for Ca dissolution compared to the same crystal facet of β-C_2_S [11]. In contrast, the (010) facet of β-C_2_S has shown an opposite trend, where, Ca-1822 was required to overcome the comparatively higher barrier of 169.60 kJ/mol and the process is endergonic (Δ*G* of +50.20 kJ/mol) and not favorable (Figure 3c).

**Figure 4:**
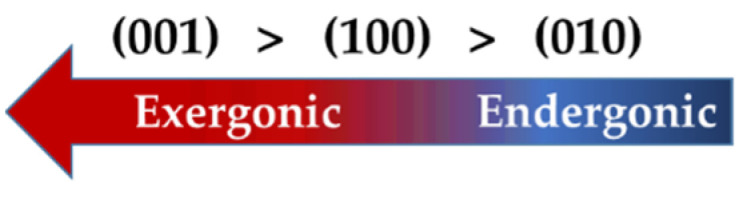
The overall order of reactivity for different facets of β-C_2_S.

Considering the thermodynamic properties, the following reactivity order for β-C_2_S is proposed in Figure 4. 

### 3.3. Dissolution Scenarios of Calcium from the First Layer of (100) Facet of β-C_2_S (Case I & Case II)

In case I, the selected Ca for simulating the dissolution process (called the central one) is positioned in between two silicates and surrounded by four neighboring Ca within a 5 Å radius, where two Ca are located in the Ca row and the other two are part of the alternating Ca-silicate row. Only the first neighbor shell of the central Ca is considered as influential, thus disregarding the next-nearest neighbors justified by the low interaction energy due to higher distance and avoiding huge expansion of the scenario possibilities.

Figure 3a represents the free energy surface for the dissolution of central Ca in-between silicates in presence of four Ca neighbors required to overcome 164.30 kJ/mol. However, introducing a crystal defect by removing one Ca neighbor placed in Ca-row only slightly reduces the activation barrier, having 154.00 kJ/mol. Additionally, the Δ*G* value of −13.80 kJ/mol indicates that this dissolution case is an exergonic process. 

**Figure 5:**
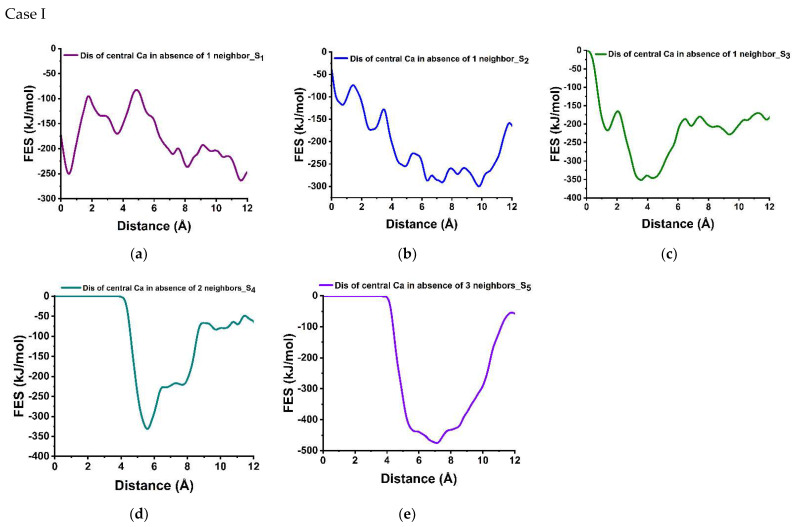

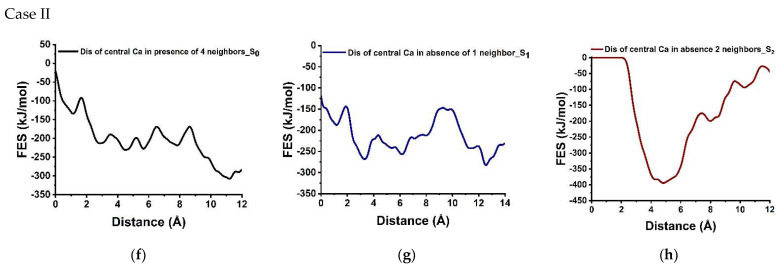
The representative dissolution profile is quantified by free energy surface (FES) of (100) facet of β-C_2_S. Case I: dissolution of central Ca surrounded by four Ca neighbors (red Ca in Figure 1I) in different scenarios: the presence of all four Ca neighbors (**a**) and stepwise absence (**b**–**e**). Case II: dissolution of central Ca located in Ca row (red Ca in Figure 1II) in the presence of all four Ca neighbors (**f**) and absence of 1,2 Ca neighbors (**g**,**h**) at 298 K.

In contrast to the missing neighbor in the Ca-row, when departing from the perfect crystal scenario by removing one neighbor Ca from the Ca-silicate row (Figure 5b), the dissolution behavior of central Ca was affected significantly resulting in a massive reduction in activation energy to 44 kJ/mol. The influence was very close to scenario 3 (Figure 5c), where the activation energy of the central Ca was calculated as 52 kJ/mol in absence of two Ca neighbors from the Ca-row. Moreover, after stepwise removal of the second neighbor (Figure 5d), the dissolution of the central Ca becomes barrierless to move spontaneously toward the solution. Likewise, with further removal of neighboring Ca (in absence of the three and four Ca neighbors) the dissolution of central Ca remains a barrierless reaction (Figure 5e).

In case II, the central Ca is located in the Ca-row. Since it does not have as strong an electrostatic interaction with the oxygens of the silicate as in case I, the dissolution of the central Ca resulted in a significantly lower activation barrier of 44.60 kJ/mol (Figure 5f), in the presence of four neighbors. The dissolution process was exergonic and thermodynamically more favorable (ΔG = −173.60 kJ/mol) compared to the Ca in case I. Following the removal of one neighboring Ca from the same row reduces the activation energy only slightly (42.40 kJ/mol), however, further removal of Ca neighbors with any combination (absence of 2, 3, 4 Ca) leads to the barrierless dissolution. The observation revealed that the activation barrier of Ca dissolution atomistic process from the Ca-row is much lower compared to the Ca-silicate row.

### 3.4. Dissolution Scenarios of Silicate from the First Layer of (100) Facet of β-C_2_S (Case III)

**Figure 6:**
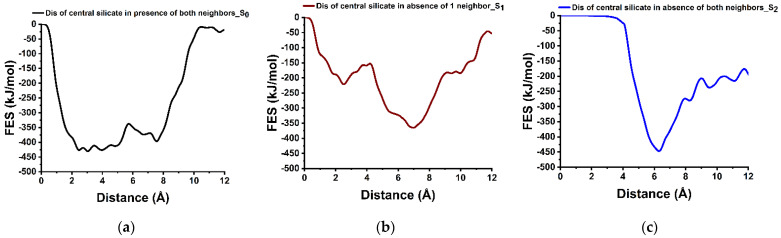
The representative dissolution profile (free energy surface) of the central silicate (marked yellow in Figure 1III, S_0_) (**a**–**c**), from (100) facets of β-C_2_S in the different scenarios: before and after removal of one and two silicate neighbors at 298 K.

The hydration of (100) facet of C_2_S is proposed to be based upon the dissolution of Ca^2+^ ions first, followed by the dissolution of SiO_4_H_4_ after protonation of all four oxygens of the silicate into the pore solution. An argument for this is that typically, it is very difficult to dissolve SiO_4_^4−^ due to the very strong electrostatic interaction between its four oxygens and the surrounding Ca. Therefore, first of all, the Ca atoms around the silicate row are to be removed to allow for pre-hydration scenario where three oxygen of the silicate are protonated, and the fourth one (located downside) still interacts with the second layer of Ca (Figure 1III). Thus, after the silicate row is made free from neighbor Ca in all three directions, a condition is met to compute the silicate dissolution. For this also the influence of silicate neighbors (linear, i.e., left and right manner) and Ca from the second layer is considered as well. The dissolution of central silicate from the (100) facet in the presence of both silicate neighbors is an endergonic process (ΔG = +31.00 kJ/mol) that requires to overcome the activation barrier of 89.00 kJ/mol (Figure 6a). Nevertheless, after removing one silicate neighbor the activation energy decreases to 67.70 kJ/mol (Figure 6b), and the dissolution of silicate becomes thermodynamically favorable (ΔG = −144.60). Furthermore, the dissolution was barrierless in absence of both neighbors (Figure 6c).

### 3.5. Dissolution Scenarios of Calcium from the Second Layer of (100) Facet of β-C_2_S (Case VI)

**Figure 7:**
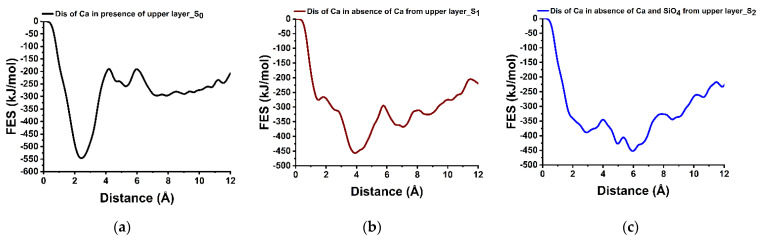
The representative dissolution profile of the Ca from the second layer (marked yellow in Figure 1IV, S_0_) (**a**–**c**), from (100) facets of β-C_2_S in the different scenarios: before and after removal of Ca and silicate from the first layer at 298 K.

In the second layer, the Ca dissolution occurs preferentially only on the active site that has created an open path through the first layer, i.e., absence of relevant nearest neighbors. The dissolution of Ca from the second layer was not possible due to a very high energy barrier of 355.70 kJ/mol while the first layer was intact. It was unfavorable toward dissolution due to the strong electrostatic interactions as well (Figure 7a). However, after removing the Ca from the first layer, the activation energy of the selected Ca is reduced to 161.90 kJ/mol; still thermodynamically unfavorable (ΔG = +89.70 kJ/mol). Further removal of the silicate located above the Ca reduced the steric hindrance and the energy barrier to 44.30 kJ/mol. The dissolution process became thermodynamically favorable (ΔG = −62.70 kJ/mol) due to easier movement through the channel created by the removal of Ca and silicate from the first layer.

### 3.6. Upscaling the Dissolution Rate for (100) Facets of β-C_2_S

The upscaling of the atomistic simulations to the meso-scopic (sub-micro) scale by the Kinetic Monte Carlo (KMC) approach is successfully implemented for the portlandite system [11,25]. A similar gap bridging concept will be used to calculate the overall rate of dissolution for (100) facets of β-C_2_S. This is now made possible by creating a catalogue of all relevant activation barriers as obtained from the dissolution of Ca and Silicate (central) sites in four different cases with all relevant scenarios by MD simulations (combination MetaD and ReaxFF) (Table 1). The rate of individual crystal site dissolutions can easily be calculated by the transition state theory [38,38] as a function of the obtained activation energies. The catalogue of the dissolution rate of all scenarios presented in this paper thus provided an input for KMC simulations, where, KMC can compute the overall (upscaled) dissolution rate of (100) facet of C_2_S in meso-scale and to investigate the morphological changes at far-from-equilibrium conditions. 

## 4. Conclusions

The foremost objective of this research is to improve the fundamental knowledge of the dissolution mechanism of belite during the early hydration of Portland cement. This paper explores the potential of the gap-bridging upscaled atomistic modeling approach. The reactivity of different crystal facets of β-C_2_S during early hydration was elucidated using ReaxFF coupled with MetaD. Moreover, for the implementation of a KMC computational upscaling approach, the atomistic forward reaction rates of Ca and silicate were studied intensively by MD simulation considering four different cases with possible scenarios depending on the neighbor configuration of (100) facet. 

The results for the β-C_2_S facets can be summarized as (100) and (001) facets are found reactive and, the dissolution of Ca is thermodynamically favorable during the initial hydration stage. However, they are less reactive compared to the C_3_S due to the absence of interactive free oxygen and higher number of silicates that have strong electrostatic interaction between the silicates’ four oxygens with the surrounding Ca. Moreover, water tessellation on the (010) facet was an influential factor to prevent Ca dissolution, thus explaining the lowest reactivity among the three facets.

The atomistic scale dissolution by considering a different type of Ca and silicate (cases) with relevant scenarios is demonstrated to be vital for upscaling using KMC simulation. The Ca located in the Ca row was (Case II) found to dissolve faster in comparison to Ca in the Ca-silicate row (Case I). In both cases the Ca dissolution are exergonic process, however, in case I the activation barrier for dissolving the central Ca was almost four times higher compared to the central Ca in case II (i.e., in presence of 4 Ca neighbors) due to the strong electrostatic interaction of oxygen of two neighboring silicates. Nevertheless, the activation barrier decreases by almost 73% in the absence of two neighbors and becomes barrierless on the further dissolution of neighbors (three and four) for Case I, and consequently, the dissolution rate increases by reducing the number of neighbors in a stepwise manner. In Case II, the reduction in the activation barrier of central Ca was 5% in the absence of 1 neighbor; however, it become barrierless in absence of two, three, and four Ca neighbors.

Silicate dissolution was not possible due to strong electrostatic interaction with Ca neighbors. Therefore, only after the removal of all surrounding Ca enabled a scenario to compute the influence of silicate neighbors. Initially, the dissolution of the central silicate was unfavorable, however, removing the first silicate neighbor resulted in the reduction in activation energy (24%), and, dissolution becomes barrierless and thermodynamically favorable in absence of both silicate neighbors.

In case IV, the dissolution of Ca from the second layer was unfavorable at the perfect crystal conditions, however, after removing the Ca and silicate from the first layer, the dissolution of Ca becomes easier through the channel created by the displacement of atoms, which indicates the possibilities for etch pit formation. 

Finally, the calculated dissolution rate of all relevant cases for crucial scenarios for (100) facet of C_2_S is made available to feed the KMC for upscaling simulation (presented in Part 2 of the paper).

## Figures and Tables

**Table 1:** Thermodynamic properties of different cases with possible dissolution scenarios of calcium and silicate from the (100) facet of β-C_2_S at 298 K.

Cases	Possible Scenarios of Dissolution of Central Ca or Silicate	Free Energy of Activation (ΔG*) kJ/mol	Free Energy Change (Δ*G*) kJ/mol	Thermodynamic Properties
I	S_0_: In presence of 4 neighbors	164.30	−78.00	Exergonic
Ca dissolution	S_1_: In absence of 1 neighbor	154.80	−13.80	Exergonic
	S_2_: In absence of 1 neighbor	44.00	−182.00	Exergonic
	S_3_: In absence of 2 neighbors	52.00	−130.16	Exergonic
	S_4_ : In absence of 2 neighbors	0.00	*	----
	S_5_: In absence of 3 neighbors	0.00	*	----
II	S_0_: In presence of 2 neighbors	44.60	−173.60	Exergonic
Ca dissolution	S_1_: In absence of 1 neighbor	42.40	−94.60	Exergonic
	S_2_: In the absence of 2 neighbors	0.00	*	----
III	S_0_: In presence of 2 neighbors	89.00	+31.00	Endergonic
Silicate	S_1:_ In absence of 1 neighbor	67.70	−144.60	Exergonic
dissolution	S_2_: In the absence of 2 neighbors	0.00	*	----
IV	S_0_: In presence of upper layer Ca	355.70	+247.70	Endergonic
Ca dissolution from 2nd layer	S_1_: In absence of upper layer Ca	161.90	+89.70	Endergonic
	S_2_: In absence of upper layer Ca and one silicate	44.30	−62.70	Exergonic

* barrierless.

## Abbreviations

**MD**	**Molecular Dynamics**
C2S	Belite, Dicalcium Silicate, 2CaO SiO2
C3S	Alite, Tricalcium Silicate, 3CaO SiO2
KMC	Kinetic Monte Carlo
ReaxFF	Reactive Force Field
LAMMPS	Large-scale Atomic/Molecular Massively Parallel Simulator
PLUMED	PLUgin for MolEcular Dynamics
MetaD	Metadynamics
CVs	Collective Variables
TS	Transition State
VNL	Virtual Nano Lab
FES	Free Energy Surfaces (kJ/mol)
HFTN	Hessian-free truncated Newton algorithm
NVT	Nose-Hoover thermostat
NPT	Nose-Hoover pressure barostat
Δ*G**	Free Energy of Activation Barrier (kJ/mol)
Δ*G*	Free Energy Change (kJ/mol)

## Appendix A

**Table A1:** Crystallographic data for the orthogonal simulation cells of β-C_2_S consisting of different crystalline facets at 298 K.

Crystal Facet of β-C_2_S	Orthogonal Cell Dimension (Å^3^/10^−30^ m^3^)	No. of Atoms in the Simulation Cell
(100)	23.51, 27.34, 48.99	1899
(001)	23.56, 27.38, 40.73	2141
(010)	27.75, 33.97, 47.74	3848
